# Polyethylene Glycol Coated Magnetic Nanoparticles: Hybrid Nanofluid Formulation, Properties and Drug Delivery Prospects

**DOI:** 10.3390/nano11020440

**Published:** 2021-02-09

**Authors:** Rashmi Mannu, Vaithinathan Karthikeyan, Nandakumar Velu, Chandravadhana Arumugam, Vellaisamy A. L. Roy, Anantha-Iyengar Gopalan, Gopalan Saianand, Prashant Sonar, Kwang-Pill Lee, Wha-Jung Kim, Dong-Eun Lee, Venkatramanan Kannan

**Affiliations:** 1 Department of Physics, SCSVMV Deemed University, Kanchipuram 631561, India; mrashme@gmail.com (R.M.); vnandakumar1981@gmail.com (N.V.); chandu.vadhana@gmail.com (C.A.); 2 Department of Physics, St. Joseph’s College of Arts and Science for Women, Hosur 635126, India; 3 Department of Materials Science & Engineering, City University of Hong Kong, Hong Kong; vkarthike2@cityu.edu.hk; 4 Department of Electronics & Nanoscale Engineering, University of Glasgow, Glasgow G12 8QQ, UK; roy.vellaisamy@glasgow.ac.uk; 5 Daeyong Regional Infrastructure Technology Development Center, Kyungpook National University, Daegu 41556, Korea; algopal99@gmail.com (A.-I.G.); kplee@knu.ac.kr (K.-P.L.); kimwj@knu.ac.kr (W.-J.K.); 6 Global Center for Environmental Remediation (GCER), College of Engineering, Science and Environment, The University of Newcastle, Callaghan, NSW 2308, Australia; SaiAnand.Gopalan@newcastle.edu.au; 7 School of Chemistry and Physics, Queensland University of Technology (QUT), 2 George Street, Brisbane QLD 4001, Australia; Sonar.Prashant@qut.edu.au; 8 School of Architecture, Civil, Environment and Energy Engineering, Kyungpook National University, 80, Daehakro, Buk-gu, Daegu 41566, Korea

**Keywords:** magnetic nanofluids, drug delivery, drug release models, susceptibility

## Abstract

Magnetic nanoparticles (MNPs) are widely used materials for biomedical applications owing to their intriguing chemical, biological and magnetic properties. The evolution of MNP based biomedical applications (such as hyperthermia treatment and drug delivery) could be advanced using magnetic nanofluids (MNFs) designed with a biocompatible surface coating strategy. This study presents the first report on the drug loading/release capability of MNF formulated with methoxy polyethylene glycol (referred to as PEG) coated MNP in aqueous (phosphate buffer) fluid. We have selected MNPs (NiFe_2_O4, CoFe_2_O4 and Fe_3_O_4_) coated with PEG for MNF formulation and evaluated the loading/release efficacy of doxorubicin (DOX), an anticancer drug. We have presented in detail the drug loading capacity and the time-dependent cumulative drug release of DOX from PEG-coated MNPs based MNFs. Specifically, we have selected three different MNPs (NiFe_2_O_4_, CoFe_2_O_4_ and Fe_3_O_4_) coated with PEG for the MNFs and compared their variance in the loading/release efficacy of DOX, through experimental results fitting into mathematical models. DOX loading takes the order in the MNFs as CoFe_2_O_4_ > NiFe_2_O_4_ > Fe_3_O_4_. Various drug release models were suggested and evaluated for the individual MNP based NFs. While the non-Fickian diffusion (anomalous) model fits for DOX release from PEG coated CoFe_2_O_4_, PEG coated NiFe_2_O_4_ NF follows zero-order kinetics with a slow drug release rate of 1.33% of DOX per minute. On the other hand, PEG coated NiFe_2_O_4_ follows zero-order DOX release. Besides, several thermophysical properties and magnetic susceptibility of the MNFs of different concentrations have been studied by dispersing the MNPs (NiFe_2_O_4_, CoFe_2_O_4_ and Fe_3_O_4_) in the base fluid at 300 K under ultrasonication. This report on the DOX loading/release capability of MNF will set a new paradigm in view that MNF can resolve problems related to the self-heating of drug carriers during mild laser treatment with its thermal conducting properties.

## 1. Introduction

In recent times, there has been a gradual increase of interest in developing newer nano-systems for diverse biomedical applications such as photoablation therapy, biosensors, hyperthermia, bio-imaging and targeted drug delivery [1]. Nanotechnology can be effectively used to circumvent a few of the drawbacks of conventional drug loading/release formulations. Magnetic metal oxide-based nanoparticles (typically iron oxide) are unique due to their exceptional chemical, biological, catalytical and magnetic properties along with chemical stability, non-toxicity, biocompatibility, elevated saturation magnetization and appropriate magnetic susceptibility [2,3,4,5,6,7,8,9,10,11,12,13,14,15,16]. These properties form the basis for their biomedical applications [17,18,19,20,21,22,23]. While iron oxides are the most prevalently used magnetic nanoparticles (MNPs), alloys such as Fe-Co, Fe-Ni, as well as metal ferrates such as CoFe_2_O_4_ and MnFe_2_O_4_ NPs are being explored for use in biomedical applications as conceivable substitutes to iron oxide NPs [3]. Newer MNPs were designed and developed by doping magnetically susceptible materials such as manganese (Mn), cobalt (Co) and nickel (Ni) into iron oxide NPs tailor-designed to impart additional functionalities such as magnetic susceptibility, etc. [24]. Magnetic nanofluids (MNF) or ferrofluids are obtained by dispersing MNPs such as metallic Fe, metallic Co, Fe_3_O_4_, Fe_2_O_3_, CoFe_2_O_4_, etc. in a base liquid like water, ethylene glycol, kerosene, etc. [25]. The physicochemical properties of ferrites can be suitably modified to extend their potential prospects in sensors, catalysts, adsorbents, enzyme supports, ferrofluid technology, targeted drug delivery, tissue engineering, local hyperthermia and contrast agents in nuclear magnetic resonance imaging [26]. MNFs can be used as drug delivery vehicles for cancer patients. MNPs are excellent aspirants for cancer therapy as they can absorb more magnetic power than microparticles in alternating current magnetic fields and they are more adhesive to tumor cells than non-malignant cells [27]. Compared to other noble metal-type NPs, the magnetic NPs afford distinguishing characteristics for the handling of the NF by magnetic force [28,29,30,31,32,33,34,35]. The MNF containing magnetic NPs absorbs energy in an alternating electromagnetic field and acts as a super-paramagnetic fluid. Hyperthermia can yield a favored radiation effect on malignant cells, due to the enhanced chemotherapeutic efficiency [36]. The superparamagnetism of MNPs is generated by thermal effects and strong enough to spontaneously demagnetize the superparamagnetic assembly. The coercivity of MNPs will become zero and no hysteresis is possible. The superparamagnetic NPs exhibits magnetic properties in the presence of an external magnet but revert into a non-magnetic state upon removing the magnetic field. This behavior of superparamagnetic materials is important for drug delivery therapeutics usage onto specific sites. 

We believe that MNFs with adequate coating would enhance these characteristics with their inbuilt thermal conducting properties. The colloidal stability of water based MNF and surface modification of NPs are the two most significant aspects that favor fruitful application [37]. Nanosized ferrites have been synthesized by many researchers by techniques like pulsed wire discharge method, reverse micelle technique, co-precipitation method, emulsion method, hydrothermal method and high energy ball milling, etc. Nickel ferrites (NiFe_2_O_4_) are widely used in high-frequency applications like microwave due to their adequately low hysteresis losses and high resistivity [38]. NiFe_2_O_4_ is a soft ferromagnetic material with all nickel ions located in the B-sites and ferric ions in both A-sites and B-sites crystallizing with a completely inverse and cubic spinel structure [39]. Nanocrystalline NiFe_2_O_4_ particles were synthesized using simple self-combustion techniques with the help of albumen, which plays the role of fuel in the combustion process [40]. CoFe_2_O_4_ is the most suitable candidate for biomedical applications, as it has excellent physical and chemical stability, tunable coercivity, saturation magnetization, large anisotropy property and interesting inverse spinel cobalt ferrite structure [41]. The antimicrobial activity of the CoFe_2_O_4_ NPs on pathogenic and multidrug-resistant bacterial strains has also been reported [42]. The synthesis, characterization and magnetic properties of CoFe_2_O_4_ nanorods have also been studied as well as its application in the medical field [43]. Fe_3_O_4_ NPs have been greatly used in many medical applications because of their excess biosafety, readymade availability, governable features compared to other NPs [44]. Magnetic nano reagents (MNR) comprised of bio probe-coated MNP and liquid solvents have been established to progress the medical functions as compared to the other current medical procedures like ELISA [45]. Many strategies have been carefully devised and examined to obtain sub-nano magnetic particles through micro-emulsion and poly-process and chemical co-precipitation, which is extremely popular because of its large-scale production potential, simplicity and inexpensive nature [46]. 

In this report, the thermo-physical properties and biomedical applications of a few of the MNFs (NiFe_2_O_4,_ CoFe_2_O_4_ and Fe_3_O_4_) are analyzed. It is important to note that the utilization of MNPs in a biomedical application requires judicious control of particle size, non-fouling surfaces, and increased functionalities. Especially, MNPs with adequate biofunctionalization are required for drug loading/release application. Importantly, surface modification of MNPs can be effective for multifunctional prospects such as diagnosis and therapeutic utility. The development of MNPs with appropriate surface functionalization has advanced exponentially over the past few years. In this study, magnetic NPs are coated with polyethylene glycol (PEG). To our knowledge, the efficacy of MNFs formulated with PEG-coated MNPs has not been evaluated for drug loading and release characteristics. We have used the anti-cancer drug, doxorubicin hydrochloride (referred to as DOX), as the model drug for demonstrating the drug loading/release capabilities of the formulated MNF with aqueous phosphate buffer as the base liquid.

## 2. Materials and Methods

### 2.1. Materials

Magnetic NPs (NiFe_2_O_4_, CoFe_2_O_4_ and Fe_3_O_4_) used in the present study were procured from US Nano Laboratories (Houston, TX, USA) and used as received. Methoxy polyethylene glycol (referred simply as PEG), 3-aminopropyl triethoxysilane (APTS) were obtained from Axiom Chemicals Private Limited (Gujarat, India) and dimethylformamide (DMF) was purchased from Sonia Industries (Delhi, India).

### 2.2. Modification of MNP with mPEG

**Scheme 1:**
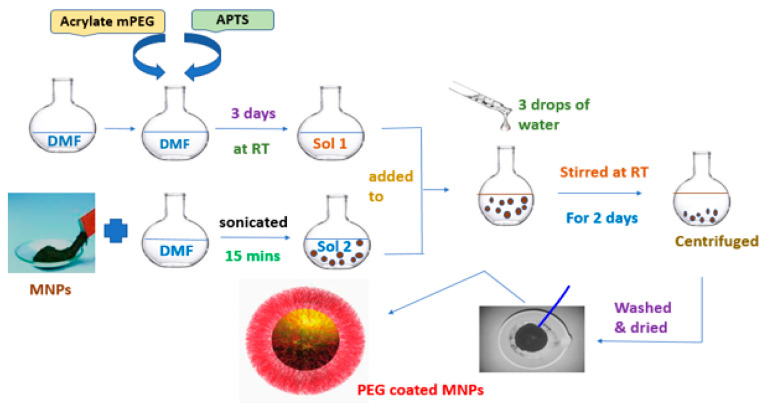
Modification of magnetic nanoparticles (MNPs) with polyethylene glycol (PEG) coating.

The procedure carried out for the modification of magnetic NPs with PEG involves two steps, as given in Scheme 1. In the first step, PEG (2.2 g, 2 mmol) and APTS (0.45 mL, 2 mmol) were dispersed in DMF and the mixed solution was kept for 3 days at room temperature. In the second step, MNPs (0.5 g) were dispersed in DMF (25 mL), then sonicated for 15 min and added to the above solution. Three drops of water were added to facilitate the hydrolysis process of silane and the evolved mixture was stirred at room temperature for 2 days. The PEG Coated MNPs were obtained by centrifugation with the applied magnetic field and washed well with water and dried under a vacuum.

### 2.3. MNF Preparation

The magnetic NPs (NiFe_2_O4, COFe_2_O4 and Fe_3_O4) were dispersed in distilled water to obtain the respective NFs. Typically, 0.080 g of MNPs is dispersed in 40 mL of distilled water for the preparation of MNF in 0.2% and sonicated with a frequency of 24 kHz for about 3 h at 303 K to ensure effective dispersion of particles. Similarly, 0.160 g, 0.240 g of MNPs is dispersed in 40 mL of distilled water to prepare MNF in 0.4% and 0.6%, respectively. NiFe_2_O_4_, COFe_2_O_4_ and Fe_3_O_4_) were used independently as MNP and MNFs were prepared in ten different concentrations (0.2%, 0.4%, 0.6%, 0.8%, 1.0%, 1.2%, 1.4%, 1.6%, 1.8% and 2.0%).

### 2.4. DOX Loading/Release in MNF with PBS as the Base Fluid

#### 2.4.1. DOX Loading

**Scheme 2:**
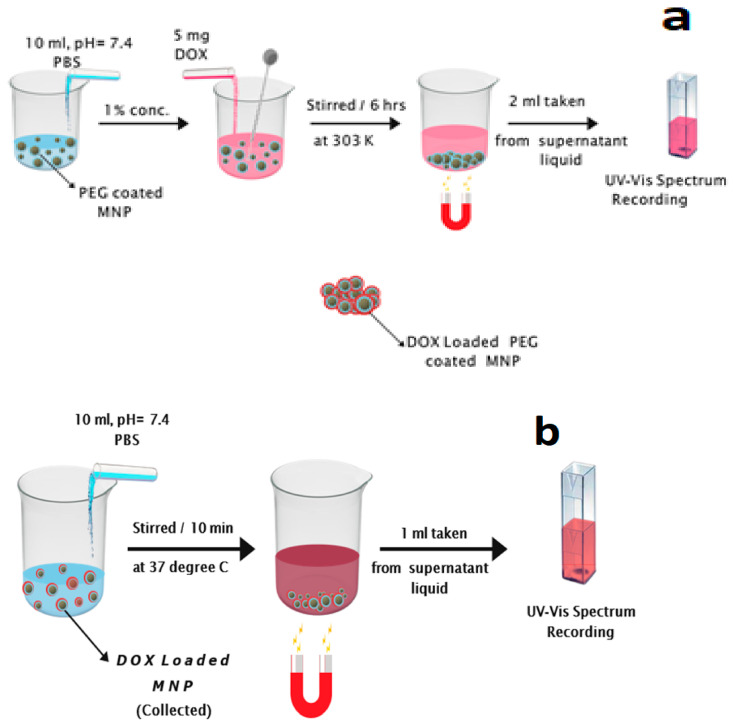
(**a**) Doxorubicin (DOX) loading and (**b**) DOX release procedure with PEG-coated MNP.

Our preliminary results demonstrated that the coating of PEG with a time lesser than 72 h resulted in less drug loading. The DOX loading was performed as follows as shown in Scheme 2a with 72 h for the coating process. Typically, the MNF (with PEG-coated 2% weight MNP) was prepared in PBS (pH = 7.4, 0.1 M) by adopting a similar procedure as described above. Knowing the solubility limitations in phosphate buffer, we initially dissolved 10mg of DOX (hydrochloride) in 5 mL of water. Subsequently, this water dissolved DOX solution (5 mL) was dropwise added to MNF prepared in phosphate buffer (5 mL) (with PEG-coated 2% weight MNP) and stirred for 6 h to improve the solubility of DOX in the buffer based MNF. The DOX-loaded MNPs were isolated at the bottom of the container using a magnetic bar. The extend of drug loading was determined by recording the UV-visible spectrum of the supernatant liquid after the removal of MNP. We used the molar absorptivity of DOX at 480 nm in the similarly prepared solution without MNP and used it for calculating the DOX loading level. The DOX-loaded MNPs were removed from the MNF and dried at room temperature for 12 h.

#### 2.4.2. DOX Release

The DOX loaded MNPs were redisposed in 10 mL of MNF (1%) prepared with PBS as the base fluid. The solution was gently stirred. About 2 mL of the aliquot was periodically removed from the above solution and MNPs were removed from the MNF. The supernatant liquid was tested for DOX released by recording the UV-visible spectrum over a period as shown in Scheme 2b. The released DOX concentration was determined using the absorbance at 480 nm.

### 2.5. Characterization

The JEOL JEM 2100 (Tokyo, Japan) was used to record high-resolution transmission electron microscopy (HR-TEM) of the magnetic NPs. JEOL, JSM-6390 LV (Peabody, MA, USA) from Oxford instruments EDX (High Wycombe, UK) was utilized for scanning electron microscopy (SEM with EDAX) to analyze the surface morphology and composition of elements of the MNPs. X’Pert Pro analytical powder X-ray diffractometer(Nottingham, UK) was used to record the XRD patterns of the MNPs. The diffraction patterns were recorded at room temperature using Cu Kα radiation (λ = 1.5406 Å) with Bragg’s angle varying from 10° to 80°. An ultrasonic interferometer (2 MHz, Mittal F-81 model(Delhi, India)) was used to measure the ultrasonic velocity of all the prepared MNFs (NiFe_2_O_4_, CoFe_2_O_4_ and Fe_3_O_4_) at 303K. Digital Viscometer (BROOKFIELD make) was used to measure the viscosity of the MNFs at four different temperatures (303, 308, 313, and 318K). The density of the as-prepared MNFs was measured using the specific gravity bottle (5 cc). With an Abbe Refractometer, refractive index studies were done. Using a Zetasizer Nano ZS90, Malvern Instruments Ltd., (Malvern, UK) Zeta potential was measured. A digital pH meter (Alpha-01 model, India) was used to measure the pH values of MNFs at 303 K. For sonication purposes, a GT Sonic professional ultrasonic cleaner operating at 40 kHz was used. A UV Visible Spectrometer (ANALYTIKJENA, Jena, Germany) was used for UV absorption measurements. Quincke’s method was used to determine the magnetic susceptibility of magnetic NFs.

## 3. Results and Discussion

### 3.1. Particle Size and Morphology of MNPs

**Figure 1:**
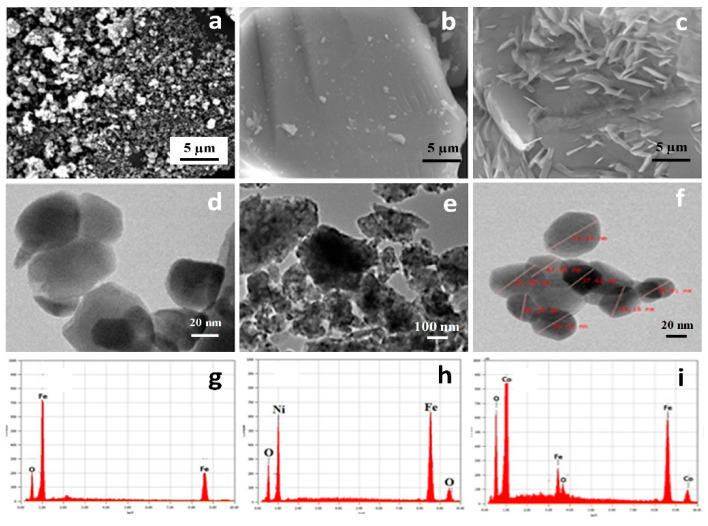
SEM images of (**a**) Fe_3_O_4_ (**b**) NiFe_2_O_4_, (**c**) CoFe_2_O_4_ NPs; TEM images of (**d**) Fe_3_O_4_. (**e**) NiFe_2_O_4_, (**f**) CoFe_2_O4 NPs; EDS image of (**g**) Fe_3_O_4_, (**h**) NiFe_2_O_4_, (**i**) CoFe_2_O_4_ NPs.

The morphology and elemental composition of MNPs (Fe_3_O_4_, NiFe_2_O_4_ and CoFe_2_O_4_) were identified by SEM coupled with EDX measurements (Figure 1). Figure 1a shows the spherical shaped Fe_3_O_4_ NPs. SEM image of NiFe_2_O_4_ NPs (Figure 1b) shows the existence of larger sized, irregularly shaped particles. A SEM image of CoFe_2_O_4_ NPs (Figure 1c) informs the presence of particles with an elongated spherical morphology. EDX analysis verifies the chemical compositions. The absence of elements other than the corresponding elements in the MNP samples, suggests that there is no contamination. A TEM image of NiFe_2_O_4_ NPs (Figure 1e) reveals the presence of random sized particles having an average particle size of 148 nm. However, TEM images of Fe_3_O_4_ and CoFe_2_O_4_ NPs (Figure 1d,f) show the narrow distribution of smaller sized spherical particles having an average size of 35 nm and 55 nm, respectively.

### 3.2. Phase Structure of Pristine MNPs and PEG-Coated MNPs

**Figure 2:**
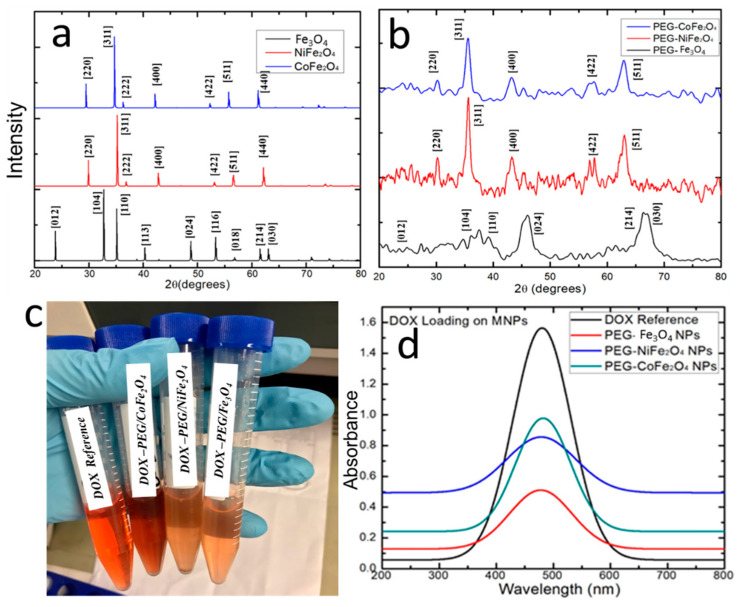
X-ray diffraction patterns of (**a**) pristine magnetic nanoparticles (MNPs) and (**b**) polyethylene glycol (PEG)-coated MNPs (Fe_3_O_4_, NiFe_2_O_4_ and COFe_2_O_4_), (**c**) doxorubicin (DOX) loaded-PEG coated magnetic nanofluid, (**d**) UV-visible absorption spectra of DOX loaded PEG-coated magnetic nanofluids (MNFs).

XRD patterns of Fe_3_O_4_, NiFe_2_O_4_ and CoFe_2_O_4_ NPs are shown in Figure 2a. The XRD pattern of Fe_3_O_4_ NPs exhibits peaks that correspond to the (220), (311), (400), (511) and (440) of the inverse cubic spinel phase (JCPDS card no. (85-1436). XRD image of NiFe_2_O_4_ consists of peaks corresponding to (200), (311), (400), (620) and (533) of a cubic spinel structure that corroborates well with the JCPDS, File No. (10-325). The XRD pattern of CoFe_2_O_4_ NPs comprises peaks that correspond to the (220), (311), (400), (511) and (440) of pure cubic structure recognized with the JCPDS, File No. (22-1086). Figure 2b shows the XRD pattern of respective PEGylation Fe_3_O_4_, NiFe_2_O_4_ and CoFe_2_O_4_ NPs.

The decrease in crystalline peak intensities suggests that PEG coating is effective on the MNPs. XRD analysis offers information about the crystalline grain size, structure, lattice parameters and phase nature of the investigated MNPs. The crystallite sizes are quantitatively determined from the XRD data using the Debye–Scherrer equation,

d = (kλ/βcos θ)(1)

where, d—particle size, θ—Bragg angle, k—Debye–Scherrer constant (0.89). It can be taken as 0.89 or 0.9 for Full-Width Half Maximum (FWHM) of spherical crystals with cubic unit cells, λ- X-ray wavelength (0.15406 nm) and β is the full width at half maximum. Calculated average crystallite sizes of Fe_3_O_4_, NiFe_2_O_4_ and CoFe_2_O_4_ are 9 nm, 38 nm and 13 nm, respectively. However, the aggregation of grains occurs to result in the formation of larger-sized particles.

### 3.3. DOX Uptake/Release Characteristics of MNFs

Exclusive characteristics of MNPs that bestow medical applications to them include biocompatibility, chemical and physical stability. When MNPs are involved in cancer therapy, one of the difficulties for them is the importance of minimizing the reaction of the medicine with the non-cancer cells. To induct tolerance of MNPs with the human body as well as for the reduction of the “efflux pumps” and transport of the drug to the outer layer of the cell, the cancer drug is to be loaded onto MNPs. DOX, the most popularly used cancer drug, can be coupled to MNPs to formulate the DOX magnetic nanocarrier [47]. DOX can create electrostatic interactions with negatively charged particles present in the MNPs or bound covalently to magnetic nanocarriers [48]. The use of MNPs as the nanocarrier is constrained with the probable van der Waals and magnetic attractions between particles, which cause aggregation of particles resulting in instability of the particles [49]. The prevention of aggregation and improvement in colloidal stability can be done with a suitable surface functionalization procedure and choice of solvent which are crucial factors for obtaining magnetic particles with sufficiently enough repulsive interactions to prevent agglomeration. The surface modification of MNPs can be effected using long-chain organic ligands or inorganic/organic polymers, either by in situ or post-modification strategies [50]. Organic polymer coating onto MNPs can result in the stabilization of MNP through steric stabilization and/or electrostatic stabilization. The selection of PEG is based on its advantageous characteristics such as hydrophilic nature, water solubility, biocompatibility, nonantigenic character and protein-resistant property. More importantly, PEGs are FDA-approved biocompatible polymer additives and demonstrated their superiority in numerous pharmaceutical formulations [51]. This study reports the use of PEG Coated MNPs for DOX loading/release applications. In this work, we are reporting the efficacy of PEG Coated MNPs based MNFs for DOX uptake and release measurements for the first time.

#### 3.3.1. DOX Loading

DOX (5 mg) solution was prepared in MNF with PBS (pH = 7.4), which was kept for 6 h for loading DOX. The DOX-loaded MNPs were drawn to the bottom of the container by a magnet. The UV-Visible spectrum of the supernatant liquid (MNF) after removal of DOX loaded MNP shows that there is a variation in the extent of loading between the different PEG Coated MNPs (NiFe_2_O_4_, CoFe_2_O_4_ and Fe_3_O_4_) (Figure 2d). The % of DOX loading by the respective MNF was determined by monitoring the residual DOX concentration in the supernatant liquid using the absorbance at 480 nm. The % DOX loading of CoFe_2_O_4_, NiFe_2_O_4_ and Fe_3_O_4_ is 68.8, 51.0 and 38.8, respectively. Hence, it is inferred that DOX loading takes the order in the MNFs as CoFe_2_O_4_ > NiFe_2_O_4_ > Fe_3_O_4_.

#### 3.3.2. DOX Release Kinetics

**Figure 3:**
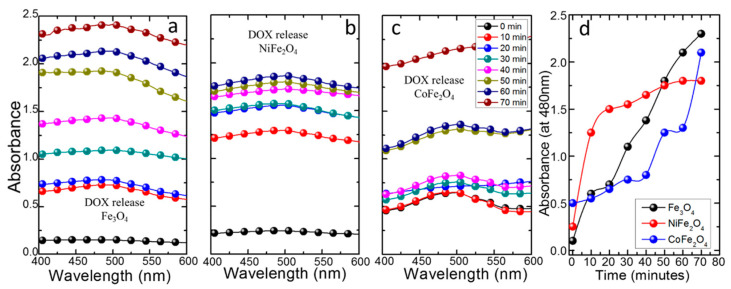
Time-dependent DOX release from the PEG coated magnetic nanoparticles based nanofluids (**a**) Fe_3_O_4_, (**b**) NiFe_2_O_4_, (**c**) CoFe_2_O_4_, (**d**) Absorbance at 480 nm.

The DOX release of the Fe_3_O_4_, CoFe_2_O_4_ and NiFe_2_O_4_ MNFs were monitored by dispersing the DOX loaded PEG coated MNPs based MNFs and recording the UV-Visible spectra throughout DOX release. as shown in Figure 3. Firstly, we would like to mention that DOX loaded PEG coated MNPs were dried and redistributed in the PBS. Hence, it could be possible that in DOX release kinetic determination at the beginning would arise from simple solubilization of the superficial DOX, followed by DOX diffusion from the matrix. The DOX release profile over time is therefore expected to involve the above phenomena at the beginning time and subsequently the drug release is expected to follow a mechanistic route as predicted by the theoretical model. The amount of adsorbed DOX on adsorbent (mg DOX/g adsorbent) was determined by knowing the absorbance values at 480 nm and correlating them to the concentration of DOX release to understand the drug release capabilities. Several mathematical models/equations can be used to understand drug release kinetics. These mathematical equations are a manifestation of the drug release profile. Generally, the drug release from the matrix on which it is loaded is controlled by various processes mainly involving a few of them such as dissolution, diffusion, partitioning, osmosis, swelling and erosion. The drug release model for the selected drug-matrix combination can be selected through the evaluation of the drug release profile by correlating with the best fit mathematical equation to define the drug release kinetics [52,53,54]. In this work, we have chosen the Zero-order kinetic model, the First-order kinetic model, the Higuchi model [52] and Korsmeyer–Peppa’s models [53,54] to analyze the DOX release data from the PEG coated MNP in the MNF. 

A brief description of the four models of choice in this work is presented. The zero-order kinetic model is designated considering that the DOX release rate is independent of its concentration [55]. According to the model, the equation

C = K_0_ t (2)

(K_0_ is the zero-order rate constant and t is the time) defines the drug release. If this model holds good, a plot of the amount of drug released versus t is expected to be linear and the drug release will be ideal with the release of the same amount of drug by a definite interval of t. With relevance to the first-order kinetic model, the drug release rate is dependent on the concentration of the drug on the matrix. The model is described by the following equation:

LogC_t_ − C_0_ = K_1_t (3)

(C_t_ − C_0_ is the amount of drug unreleased in time t and K_1_ is the first-order constant.) The linear relation between the log of cumulative of % drug unreleased and t predicts first-order drug release kinetics. The Higuchi model is represented by the equation

Q = [D (2C_0_ − C_s_)C_s_ t]^1/2^ (4)

(Q is the amount of drug released in time t, Cs is the drug solubility in the matrix media and D is the diffusivity of drug molecules in the matrix.) For data interpretation and graphical presentation, Equation (4) is modified as

Q = K_H_ t ^½^ (5)

(K_H_ is the Higuchi dissolution constant.) The linear relation between Q and t ^½^ can be correlated to the Higuchi model drug release. According to the Higuchi model, the drug release is controlled by a diffusion process based on Fick’s law. Korsmeyer and Peppa’s model is proposed to describe the empirical equation to describe both Fickian and non-Fickian release of drug from swelling as well as non-swelling polymeric delivery systems [53]. The mathematical equation for the Korsmeyer and Peppa’s model is as follows:

C_t_/C_∝_ = K t^n^ (6)

(C_t_/C_∝_ is a fraction of drug released at time t, n is diffusion exponent that indicates the mechanism of transport of drug, K is the kinetic constant.) The logarithmic form of Equation (6) can be used to infer the kinetics of drug release

Log(C_t_/C_∝_) = LogK + n Log t (7)

The linearity between Log (C_t_/C_∝_) and Log t predicts the drug release follows this model [56]. The n value of the Korsmeyer–Peppa’s models, like n ≤ 0.45 correlates for Fickian diffusion release and 0.45 < n < 0.89 for non-Fickian release (anomalous). The quasi Fickian and Fickian release correspond to the non-swellable matrix diffusion and non-Fickian release (anomalous) correlates to both diffusion and relaxation mechanism. For fixing the best model for DOX release data interpretation, the correlation coefficient (R) and coefficient of determination (R^2^), are used. Out of the two statistical parameters, the best model that describes the DOX release is predicted from R^2^ and to assess the fit of a model equation.

**Figure 4:**
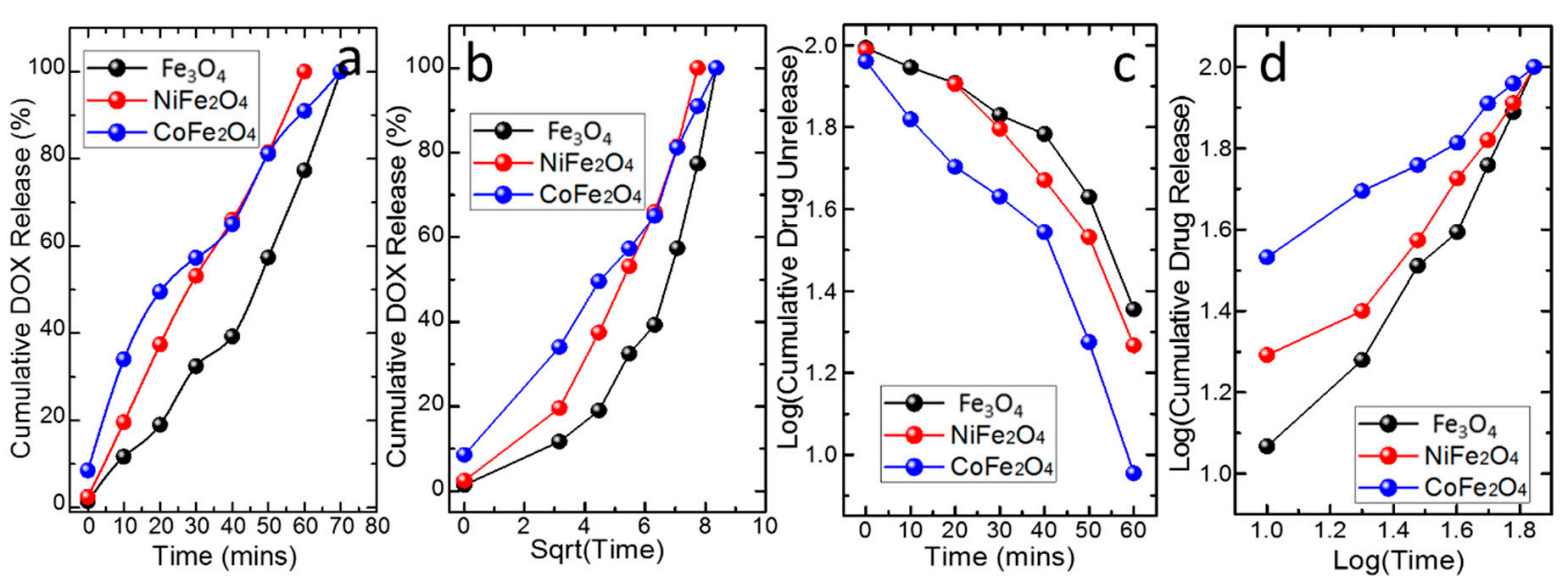
DOX release: (**a**) Zero-order, (**b**) First-order, (**c**) Higuchi and (**d**) Korsmeyer–Peppa’s kinetic model profiles of PEG Coated MNPs in PBS buffer solution.

The plots representing the cumulative DOX release % versus time, a log of cumulative drug unreleased versus time, cumulative DOX release % versus square root of time and log cumulative Dox release % versus log Time, for the PEG coated CoFe_2_O_4_, PEG coated NiFe_2_O_4_ and PEG coated Fe_3_O_4_ MNFs are shown in Figure 4 respectively.

The R and R^2^ values of the four model plots that correspond to PEG coated CoFe_2_O_4_, PEG coated NiFe_2_O_4_ and PEG coatedFe_3_O_4_ are presented in Table 1 along with the model parameters if any. The R^2^ value was used as the guideline to predict the drug release kinetics and associated mechanism. The best R^2^ for PEG coated CoFe_2_O_4_ is found to be 0.9839 (Table 1) following Korsmeyer–Peppa’s model. The n value of 0.5475 informs the predominance of non-Fickian diffusion (anomalous) for DOX release from PEG coatedCoFe_2_O_4_. The anomalous non-Fickian diffusion is correlated with the combination of both diffusions of the DOX and dissolution of PEG, which suggests the hypothesis that DOX release from PEG coated CoFe_2_O_4_ is driven by both diffusion and PEG dissolution [57].

In the case of NF based on PEG coated Fe_3_O_4_, the best R^2^ amongst the four models is 0.9727, and hence Korsmeyer–Peppa’s model is the best fit to explain DOX release. The higher n value than unity (1.1015, Table 1) informs the possible operation of a super transport II mechanism supporting the combination of relaxation and erosion [58]. The DOX release from poly (acrylic acid) coated magnetite nanoparticles followed the super transport II mechanism [59]. In the case of PEG coatedNiFe_2_O_4_, the best R^2^ is for zero-order DOX release. Generally, it is good to develop drug carriers that can have sustained or controlled drug release with a minimum dosing frequency. Drug delivery systems that follow zero-order kinetics, too, release a regular/constant/consistent amount of drug per unit time and hence are ideal for achieving prolonged drug release action [60]. Hence, the zero-order drug release profile having a constant drug release rate is preferred [61]. The DOX loaded silica–polydimethylsiloxane granules exhibited first-order drug release and the first-order kinetics has been explained based on the very small magnitude of the interfacial partition coefficient of the drug as well the small thickness of the drug depletion layer [62]. In another report on a nanoliposome-based system for dual drug delivery, zero-order release with a lower drug release rate was witnessed [63]. In the present work, DOX release from PEG coated NiFe_2_O_4_ NF follows zero-order kinetics with a slow drug release rate of 1.33% of DOX per minute. We attribute that the reservoir system maintained in PEG coated NiFe_2_O_4_ makes the DOX encapsulation through the controlling barrier membrane (PEG layer), and a zero-order mechanism was followed.

### 3.4. Thermo-Physical Properties

#### 3.4.1. Effective Velocity and Density

**Figure 5:**
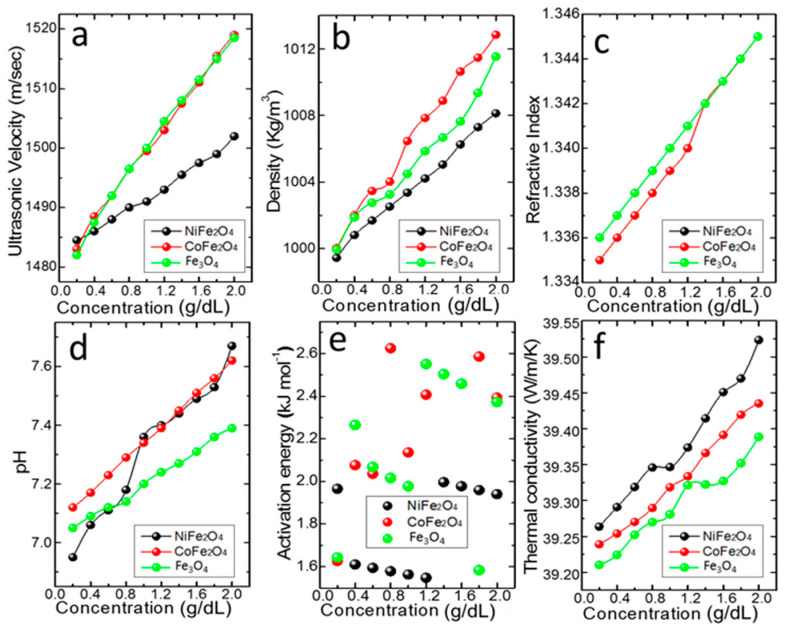
Thermophysical properties of the as-designed magnetic nanofluids. (**a**) Ultrasonic velocity, (**b**) Density, (**c**) Refractive Index, (**d**) pH, (**e**) Activation energy and (**f**) Thermal conductivity

Ultrasonic propagation in a magnetic fluid is the easiest non-destructive procedure to examine the assembly configuration exclusive from preceding changes of the sample. Ultrasonic velocity was determined for various concentrations of MNFs (NiFe_2_O_4_, CoFe_2_O_4_ and Fe_3_O_4_) at 303 K and the trend is shown in Figure 5. Ultrasonic velocity values were minimum at lower concentration (for 0.2%) and maximum at higher concentration (for 2.0%). The direct increase of velocity with concentration was observed for all the magnetic NFs. The observed increase of ultrasonic velocity in the NF was due to the presence of a molecular association between the MNPs and water molecules [64]. To designate the superficial performance, the density of all magnetic NFs (NiFe_2_O_4_, CoFe_2_O_4_ and Fe_3_O_4_), was quantified at 303 K and changes are presented in Figure 5. A linear increase in density was perceived in the three magnetic NFs for the concentration range (0.2–2.0%) [65]. NiFe_2_O_4_ NF has a minimum density compared to CoFe_2_O_4_ and Fe_3_O_4_ NF. The highest value of density is 1008.13 kg/m^3^ for NiFe_2_O_4_, 1012.84 kg/m^3^ for CoFe_2_O_4_ and 1011.54 for Fe_3_O_4_ NFs at the higher concentration range (2.0%).

#### 3.4.2. Effective Viscosity and Activation Energy

The viscosity of NF is the quantity of propensity of the dispersion to defy the flow or shear stress and shear rate ratio. It is to be noted that the increase in viscosity due to the suspension of nanoparticles is not desirable in the industry when it involves flow in micro-channel applications [66].

For the MNFs (NiFe_2_O_4_, CoFe_2_O_4_ and Fe_3_O_4_), relative viscosity (cP) values were measured at four different temperatures (303 K, 308 K, 313 K and 318 K) and given in Table 2 and Table 3. It was observed that cP increased with the concentration of MNPs and as the temperature is increased cP decreases. The decrease of cP revealed the fading of intermolecular forces owing to the thermal agitation of the NPs [67]. The increase of cP with NP weight fractions and decrease with temperature was witnessed both in the presence or absence of a magnetic field [68]. By increasing the shear rate, the agglomerates were gradually separated and the actual viscosity change decreased. The magnetorheological effect detected for the three magnetic NFs indicates the prevailing modifications in the colloidal stability of NFs [66].

Furthermore, the chemical mechanism accountable for the mass transfer such as activation energy (Q) is accounted for in the present study. The quantity of energy required to activate the atoms or molecules for a chemical reaction is known as Q. Using the Arrhenius expression, the Q was deduced [69],

η = A e^−Q/RT^ (8)

where η is the rate constant, A is the pre-exponential factor, Q is the apparent activation energy, R is the gas constant (R = 8.34 J/K/mol) and T is the temperature. Taking the logarithm of Equation (8) transforms the relation as follows,

lnη = ln A − (Q/R) 1/T (9)

The plot of ln η with the inverse of temperature (1/T) for the three MNFs gives linear plots and the slope of these curves gives the value of Q. The plot of Q against different concentrations of MNFs (NiFe_2_O_4_, CoFe_2_O_4_, and Fe_3_O_4_) is given in Figure 5e. From Figure 5, it was observed that Q of NiFe_2_O_4_ NFs decreased when concentration increased from 0.2% to 0.4%, then decreased further to 1.2% and increased at 1.4% and varied again. However, for CoFe_2_O_4_ NFs, it was increased initially to 0.4% and then suddenly decreased to 0.6% and increased at 0.8% and showed variation to 2.0%; whereas for Fe_3_O_4_ NFs, the reliance of Q was not based on weight fraction practically. A nearly linear increase and decrease in Q between 0.2% and 2.0% was noticed. The result corroborates that the NPs’ weight fraction was directly proportional to chemical reaction and activation factors while the effect of Brownian motion on concentration presented conflict in performance [70].

#### 3.4.3. Effective Refractive Index

MNFs display amazing magneto-optical properties. MNPs in the ferrofluid show uniform distribution and isotropic optical properties in the absence of an applied external magnetic field; while MNPs are aligned along the direction of the magnetic field and possess anisotropic optical properties when the magnetic field is applied [71]. Refractive index studies were made for the MNFs (NiFe_2_O_4_, CoFe_2_O_4_ and Fe_3_O_4_) at 303 K for different concentration (0.2–2.0%) and are shown in Figure 5. The value of RI was at least 0.2% by a value of 1.336 for NiFe_2_O_4_, 1.335 for CoFe_2_O_4_ NFs and 1.336 for Fe_3_O_4_ NFs, whereas the value was high at 2.0% of concentration with a value of 1.345 for NiFe_2_O_4_, CoFe_2_O_4_ and Fe_3_O_4_ NFs.

#### 3.4.4. Effective Thermal Conductivity

Several studies indicated that the addition of NPs in the base fluid caused an enhancement in thermal conductivity (k) of NFs. Thermal conductivity enhancement was witnessed with both base fluids (water and heptane) by adding Fe_3_O_4_ magnetite NPs in increasing concentration [72]. The effusivity and k enhancement of different concentrations of cobalt ferrite-based NFs were studied in the presence of an applied magnetic field [73] and it has been inferred that factors like NP size, morphology, pH, base fluid nature, temperature, etc., influence the k of NFs [74,75]. The k values were calculated for MNFs (NiFe_2_O_4_, CoFe_2_O_4_ and Fe_3_O_4_) using the mathematical relation,

k = 3 (N/V)^2/3^ K (10)

where k is thermal conductivity, V is the velocity of MNFs and N is Avogadro’s number. As the concentration of the MNPs increased, the thermal conductivity of the three MNFs increased [76]. The major key factor for the tremendous increment in k is the Brownian motion of the dispersed NPs. Besides, the augmented k is greatly influenced by the sonication time. Figure 5 shows that the k of NiFe_2_O_4_ NF is maximum as compared to CoFe_2_O_4_ and Fe_3_O_4_ NFs. Morphology and greater weight fraction of the MNPs resulted in the enhancement of k of the MNFs. A few typical reported pieces of evidence are presented here. The k of the NiFe_2_O_4_/Water NF (0.2%, 0.4%, 0.6%, 0.8%, and 1%) was measured at 300 K in the presence of a magnetic field (0 G to 150 G) and found to increase with the increase in a weight concentration of NF and strength of the applied magnetic field. The rise of k was more at low magnetic field strength [77]. MNFs comprising MFe_2_O_4_ (M = Fe and Co) NPS were dispersed in deionized water at different weight fractions between 0% and 4.8% and the k was determined both during the presence and absence of the magnetic field with the range of 0–500 G. Results revealed that before attaining its saturation point, by increasing the concentration and magnetic field intensity, k of MNFs showed an increasing trend [78].

#### 3.4.5. Effective Stability and pH

The rate of particle movement is profoundly reliant on the size of aggregates. Thus, it is essential to study the consequence of colloidal stability proceeding thermal physical properties and convective transfer of heat in the MNFs. The stability examination is a vital topic underneath the NF properties for many applications based on its stability nature. Measured zeta potential values suggest the stability of MNFs. Dispersions with maximum zeta potential, whether it is positive or negative, are electrically stabilized, whereas those with minimum zeta potentials lean to coalesce. Conventionally, a value of + or −25 mV is chosen to be the subjective range that parts between high and low charged surfaces [79]. Obtained zeta potential values for the present study at 1.0% concentration are −51.10 mV for NiFe_2_O_4_, −200.00 mV for CoFe_2_O_4_ and +200.00 mV for Fe_3_O_4_ NF. The pH values of the MNFs were found to have a linear relation with concentration Figure 5. It is understood that the pH of NFs is higher when compared with the base fluid (distilled water). It reveals that k and stability of the NFs were based on their pH values. The higher pH value caused an increase in k and greater stability of the NFs [80]. The easy synthesis technique, low toxicity, physical, chemical and magnetic properties, of the ferrites, are special aspects to mention [81].

#### 3.4.6. Effective Magnetic Susceptibility

Magnetic susceptibility (X) denotes the degree of magnetization of a material when subjected to an applied magnetic field. It is also known as the magnetizability of material indicating the proportion of magnetic moment and magnetic flux density. The magnetic susceptibility of liquids and liquefied gases were measured from Quincke’s method. The susceptibility of magnetic NFs (Fe_3_O_4_, NiFe_2_O_4_ and CoFe_2_O_4_) at different concentrations were determined using Quincke’s method. In Quincke’s method, the formulated MNF was taken in a U-shaped vertical tube consisting of two limbs (a wider and narrower) and kept between the pole pieces of the electromagnet. When there was no applied field, the liquid level in the narrow limb was found in the line of the center of the pole pieces of the electromagnet. When the electric field is applied, a rise of liquid in the vertical tube is observed if the MNF is paramagnetic. This is due to the action of a strong electric field at the upper surface of the vertical narrow limb and a weak electric field at the lower surface of the narrow limb. The values of magnetic susceptibility of the MNFs with concentration are presented in Table 4.

The susceptibility of the NF is calculated from the equation,

(11) $${\;X}_{2}-X_{1}=\frac{2\mathrm{gh}\left[\rho -\sigma \right]}{\mu_{0}H_{m}^{2}}$$

where X_2_—Susceptibility of the MNFs, X_1_—Susceptibility of air, ρ—density of MNFs, σ—the density of air, g—the acceleration due to gravity, h—the rise of the liquid surface in the narrow limb and H_m_—the final applied field.

When X_1_ = 0, Equation (11) becomes,

(12) $$X_{\mathrm{soln}}=\frac{2\mathrm{gh}\left[\rho -\sigma \right]}{\mu_{0}H_{m}^{2}}$$

It was observed that the X of MNFs was positive, indicating paramagnetic behavior and the base fluid, distilled water, was diamagnetic [82]. Furthermore, its physical properties could be affected by magnetic field applications. It was also found that X of MNFs (Fe_3_O_4_, NiFe_2_O_4_ and CoFe_2_O_4_) shows non-linearity with concentration due to normal and abnormal saturation. The values of X were found to be higher for Fe_3_O_4_ than NiFe_2_O_4_ and CoFe_2_O_4_.

## 4. Conclusions

In this work, we demonstrated that magnetic nanofluids formulation by choosing magnetic nanoparticles such as NiFe_2_O_4_, CoFe_2_O_4_ and Fe_3_O_4_ and efficient drug loading/release prospects can be achieved through the use of biopolymer (polyethylene glycol) surface-modification. The DOX loading/release capacity of the Fe_3_O_4_, CoFe_2_O_4_ and NiFe_2_O_4_ based MNFs showed variations among them and our results revealed that factors such as Fickian/non-Fickian diffusion, dissolution of coating matrix, drug adsorption, interfacial partition, relaxation vary depending on the magnetic nanoparticle-coating matrix–drug- combinations. These conclusions could be deduced by fitting the experimental results with theoretical models. We believe that this study provides a new avenue for extended research on the concept of nanofluid based drug release.

## Figures and Tables

**Table 1:** R^2^ values and derived parameters of the respective models.

Drug Released	Kinetic Models with Derived Parameter				
Medium	Parameters	Zero-Order	First-Order	Higuchi Model	Korsmeyer–Peppa’s Model
Polyethylene glycol (PEG) Coated	R^2^	0.9719	0.9718	0.9735	0.9839
CoFe_2_O_4_	Coefficient	0.9858	−0.9858	0.9867	0.9919
	Slope, n	1.2239	−1.2239	10.9105	0.5475
	Intercept, log K	-	-	-	0.9723
	K	-	-	-	9.3834
PEG Coated	R^2^	0.9623	0.9623	0.806	0.9727
Fe_3_O_4_	Coefficient	0.9809	−0.9809	0.8978	0.9862
	Slope, n	1.3575	−1.3575	11.0658	1.1015
	Intercept, log K	-	-	-	−0.0987
	K	-	-	-	0.7966
PEG Coated	R^2^	0.9897	0.9897	0.896	0.9688
NiFe_2_O_4_	Coefficient	0.9948	−0.9948	0.9465	0.9843
	Slope, n	1.3342	−1.3342	11.0431	0.8249
	Intercept,	-	-	-	0.4275
	log K				
	K	-	-	-	2.6765

**Table 2:** Values of relative viscosity of NiFe_2_O_4_, CoFe_2_O_4_ and Fe_3_O_4_ nanofluids (NFs).

Concentration (%)	Relative Viscosity (cP)					
	303 K			308 K		
	NiFe_2_O_4_	CoFe_2_O_4_	Fe_3_O_4_	NiFe_2_O_4_	CoFe_2_O_4_	Fe_3_O_4_
0.2	0.83	1	1	0.82	0.99	0.99
0.4	1.01	1.02	1.02	1	1.01	1.01
0.6	1.02	1.04	1.04	1.01	1.03	1.03
0.8	1.03	1.06	1.06	1.02	1.05	1.05
1	1.04	1.07	1.07	1.03	1.06	1.06
1.2	1.05	1.09	1.09	1.04	1.07	1.07
1.4	1.06	1.11	1.11	1.05	1.1	1.1
1.6	1.07	1.13	1.13	1.06	1.12	1.12
1.8	1.08	1.14	1.14	1.07	1.13	1.13
2	1.09	1.16	1.16	1.08	1.15	1.15

**Table 3:** Values of relative viscosity of NiFe_2_O_4_, CoFe_2_O_4_ and Fe_3_O_4_ NFs.

Concentration (%)	Relative Viscosity (cP)					
	313 K			318 K		
	NiFe_2_O_4_	CoFe_2_O_4_	Fe_3_O_4_	NiFe_2_O_4_	CoFe_2_O_4_	Fe_3_O_4_
0.2	0.81	0.98	0.98	0.8	0.97	0.97
0.4	0.99	1	1	0.98	0.98	0.98
0.6	1	1.02	1.02	0.99	1	1
0.8	1.01	1.03	1.03	1	1.01	1.01
1	1.02	1.04	1.04	1.01	1.03	1.03
1.2	1.03	1.06	1.06	1.02	1.04	1.04
1.4	1.04	1.08	1.08	1.02	1.06	1.06
1.6	1.05	1.1	1.1	1.03	1.08	1.08
1.8	1.06	1.1	1.1	1.04	1.09	1.09
2	1.07	1.13	1.13	1.05	1.11	1.11

**Table 4:** Variation of magnetic susceptibility of magnetic NFs with concentration.

Concentration (%)	Magnetic Susceptibility (X_m_ × 10^−10^)		
	NiFe_2_O_4_	CoFe_2_O_4_	Fe_3_O_4_
0.2	1.2885	0.0558	0.9936
0.4	1.1293	0.1120	1.3034
0.6	1.4631	0.1619	1.6317
0.8	1.0682	0.0581	0.3617
1	1.8417	0.1299	13.174
1.2	0.1801	0.0622	0.3638
1.4	0.1345	0.067	0.6257
1.6	0.1553	0.0207	0.9722
1.8	0.1102	0.0105	0.6037
2	0.0259	0.2051	0.822
